# Long non-coding RNAs and latent HIV – A search for novel targets for latency reversal

**DOI:** 10.1371/journal.pone.0224879

**Published:** 2019-11-11

**Authors:** Wim Trypsteen, Cory H. White, Amey Mukim, Celsa A. Spina, Ward De Spiegelaere, Steve Lefever, Vicente Planelles, Alberto Bosque, Christopher H. Woelk, Linos Vandekerckhove, Nadejda Beliakova-Bethell

**Affiliations:** 1 HIV Cure Research Center, Department of Internal Medicine, Ghent University and Ghent University Hospital, Ghent, Belgium; 2 Faculty of Medicine, University of Southampton, Southampton, Hants, United Kingdom; 3 San Diego VA Medical Center and Veterans Medical Research Foundation, San Diego, CA, United States of America; 4 Department of Pathology, University of California San Diego, La Jolla, CA, United States of America; 5 Department of Morphology, Faculty of Veterinary Sciences, Ghent University, Ghent, Belgium; 6 Center for Medical Genetics, Ghent University, Ghent, Belgium; 7 Division of Microbiology and Immunology, Department of Pathology, University of Utah School of Medicine, Salt Lake City, UT, United States of America; 8 Department of Microbiology, Immunology and Tropical Medicine, The George Washington University, Washington, DC, United States of America; 9 Department of Medicine, University of California San Diego, La Jolla, CA, United States of America; Florida Atlantic University, UNITED STATES

## Abstract

The latent cellular reservoir of HIV is recognized as the major barrier to cure from HIV infection. Long non-coding RNAs (lncRNAs) are more tissue and cell type-specific than protein coding genes, and may represent targets of choice for HIV latency reversal. Using two *in vitro* primary T-cell models, we identified lncRNAs dysregulated in latency. *PVT1* and *RP11-347C18*.*3* were up-regulated in common between the two models, and *RP11-539L10*.*2* was down-regulated. The major component of the latent HIV reservoir, memory CD4+ T-cells, had higher expression of these lncRNAs, compared to naïve T-cells. Guilt-by-association analysis demonstrated that lncRNAs dysregulated in latency were associated with several cellular pathways implicated in HIV latency establishment and maintenance: proteasome, spliceosome, p53 signaling, and mammalian target of rapamycin (MTOR). *PVT1*, *RP11-347C18*.*3*, and *RP11-539L10*.*2* were down-regulated by latency reversing agents, suberoylanilide hydroxamic acid and Romidepsin, suggesting that modulation of lncRNAs is a possible secondary mechanism of action of these compounds. These results will facilitate prioritization of lncRNAs for evaluation as targets for HIV latency reversal. Importantly, our study provides insights into regulatory function of lncRNA during latent HIV infection.

## Introduction

In the present era of combination anti-retroviral therapy (cART), the latent cellular reservoir of HIV is recognized as the major barrier to a cure [1–3]. Existing latency reversing agents (LRAs) are suboptimal to induce a sustained reduction of the latent reservoir *in vivo* and suffer from lack of specificity for HIV [4]. Long non-coding RNAs (lncRNAs) may present targets of choice for HIV latency reversal because they are more tissue and cell-type specific than protein coding genes [5] and can be accurately targeted by oligonucleotides.

Though the role of individual lncRNAs in regulation of HIV expression and their possible contribution to HIV latency control has been recognized, the number of lncRNAs that were studied in this setting is limited. For example, siRNA-mediated knockdown or CRISPR-Cas9-induced knockout of Nuclear Paraspeckle Assembly Transcript 1 (*NEAT1*) resulted in enhanced HIV replication and an increase of unspliced and singly-spliced HIV RNA in the cytoplasmic fraction [6, 7], consistent with a possible role of *NEAT1* in HIV RNA nuclear retention. Although these experiments were performed using productively infected cell lines [6], the results from this study suggested a possible role for *NEAT1* in post-transcriptional regulation of HIV latency via nuclear retention of HIV transcripts, warranting further investigation in appropriate model systems. Another example is Non-Protein Coding RNA, Repressor of NFAT (*NRON*), whose function in HIV latency control was demonstrated using an *in vitro* model of HIV latency and cells from HIV-infected patients receiving cART [8]. *NRON* promoted HIV latency by recruiting the transactivator protein Tat to the proteasome for degradation; knockdown of *NRON* resulted in reactivation of the latent provirus [8]. In addition, *NRON* may also regulate HIV replication via cytoplasmic retention of nuclear factor of activated T-cells (NFAT) [9], which enhances HIV transcription in primary CD4+ T-cells [10]. In contrast, *MALAT1* and uc002yug.2 lncRNAs were shown to be positive regulators of HIV replication [11, 12]. *MALAT1* sequestered polycomb repressive complex 2 from the HIV LTR, promoting its transcriptionally active state [12]. Uc002yug.2 functioned via up-regulation of Tat and down-regulation of HIV transcriptional repressors Runx1b and Runx1c; overexpression of uc002yug.2 in cells from HIV-infected patients on cART improved HIV reactivation following treatment with phytohemagglutinin M [11]. Because lncRNAs represent a greater fraction of the transcribed human genome than protein coding genes [13], it is plausible to hypothesize that there are more lncRNAs than currently demonstrated that participate in regulation of HIV expression and may contribute to HIV latency.

To-date, very few studies have attempted to explore the complexity of host-HIV interactions in the context of lncRNA expression and function. We and others have previously profiled the entire transcriptome, including lncRNAs, at different time points following HIV infection in the SupT1 cell line [14, 15]. Peng and colleagues demonstrated that early response to HIV infection included changes in expression of many lncRNA, some of which were independent of active HIV replication [15]. We further expanded on this study to explore changes in lncRNA expression during different stages of the HIV replication cycle, including reverse transcription, integration and particle production [14]. Guilt-by-association (GBA) analysis demonstrated that dysregulated lncRNAs were functionally linked to many pathways involved in regulation of T-cell function and anti-viral responses [14, 15], consistent with the idea that lncRNAs represent a part of the host response to HIV infection and may be involved in regulation of induced cellular pathways.

To our knowledge, lncRNAs have not been previously profiled during latent HIV infection in primary CD4+ T cells. In the present study, we sought to identify lncRNA dysregulated in two relevant primary T-cell models of HIV latency using RNA-Seq. The models that use replication-competent wild type HIV virus were selected in order to recapitulate more closely the effects of latency *in vivo*. Because latency is preferentially established in memory compared to naïve cells, we further aimed to determine the relationship between expression levels of lncRNAs dysregulated in latency and CD4+ T-cell maturation state. To infer the function of dysregulated lncRNA in HIV latency establishment and maintenance, we performed GBA analysis that identifies cellular pathways associated with lncRNAs. Finally, we aimed to determine whether the selected dysregulated lncRNAs are modulated by small molecule LRAs suberoylamilide hydroxamic acid (SAHA) and Romidepsin (RMD), and whether effects of SAHA and RMD on lncRNA may represent novel secondary mechanisms of action of these compounds with respect to HIV reactivation. The main motivation behind the present study was prioritization of candidate lncRNAs for further formal evaluation as targets for therapies aimed at eradication of the latent HIV reservoir.

## Results

### Models of HIV latency

**Fig 1:**
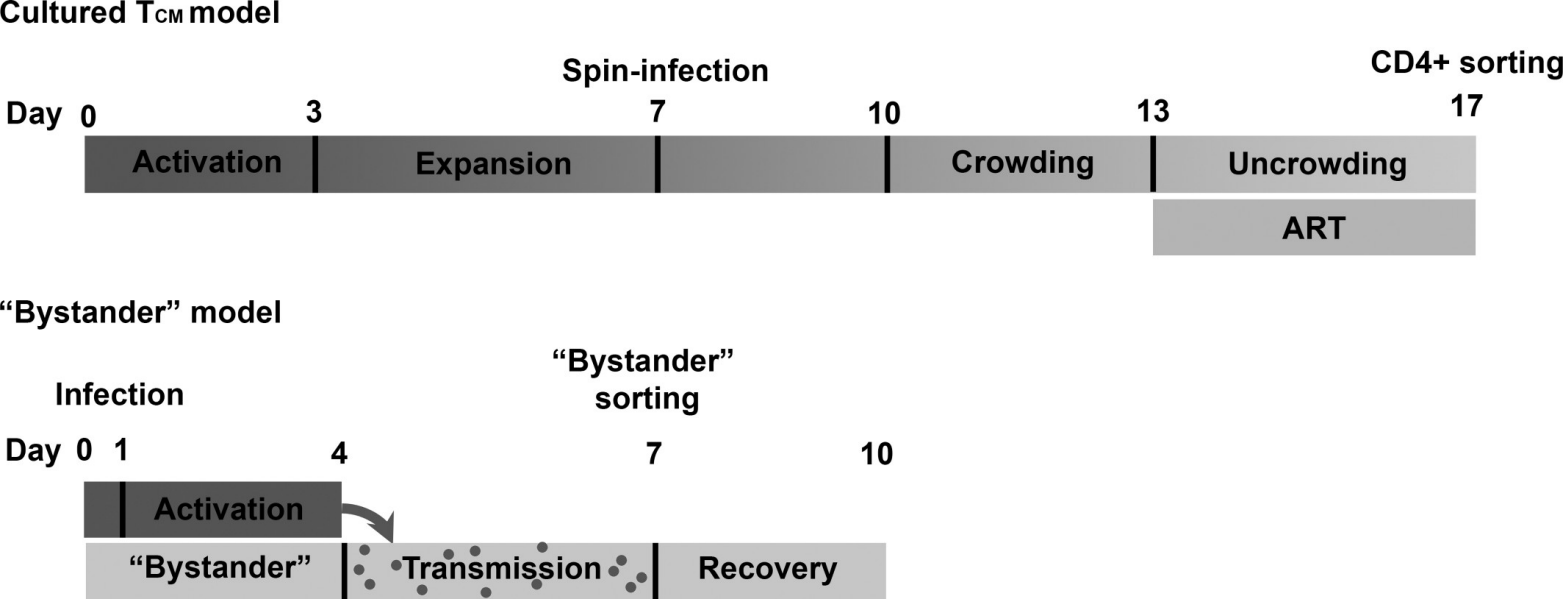
Comparison of the time-line of latency establishment between cultured T_CM_ and bystander models. Cells during activation stage are depicted with darker grey color. Resting cells are presented in light grey. Gradient of color for the cultured T_CM_ model represents gradual return from activation to quiescence. Major steps during model set-up (such as infection and sorting) are indicated above the time-line.

To identify lncRNAs dysregulated in HIV latency, the cultured T_CM_ [16, 17] and the bystander model [18, 19] of HIV latency were used. These models differ by the phenotypic composition of CD4+ T-cells and the route of latency establishment (dividing cells returning to quiescence or resting cells). Cultured T_CM_ model originally developed by Bosque and Planelles [16, 17] is established over 17 days of culture, where initial infection is conducted by spinoculation after 7 days following cell activation and expansion. Further virus replication and transmission is facilitated via cell-to-cell contact during the crowding stage till day 13, when the peak of p24+ expression is observed (21.8 ± 7.8 percent p24+ cells, N = 4). Virus spread is stopped at this point by adding antiretrovirals, and cells are allowed to return to quiescence for another 4 days [20]. Before samples are processed for sequencing, positive magnetic isolation of CD4+ T cells is used to sort out cells that may be productively infected [21]. Integration events in this model were characterized previously using Alu-PCR [21] detecting 33 ± 10.6% cells with HIV provirus on day 17, on average (N = 5), assuming 160 cells generate 1 ng DNA. “Bystander” model, described in [18] and [19], uses a shorter culture period, where a subset of cells is initially stained with a viable dye, e-Fluor 670, infected with HIV and activated, followed by mixing of these cells with resting “bystander” cells. Resting bystander cells are represented by all major phenotypic subsets found *in vivo*, including naïve, central memory and effector memory cells. Virus transmission from infected proliferating to bystander cells occurs in this co-culture via cell-to-cell contact. Peak of infection (average 37.3 ± 10.4 percent of p24+ proliferating cells, N = 4) is achieved on day 7. At this point, the resting (e-Fluor 670 negative) bystander cells are sorted out from the co-culture by fluorescence activated cell sorting. Following separation from productively infected cells, resting cells are left in culture for another 3 days to complete integration events of virus that entered the cells. Integration events in the end of culture were evaluated previously by droplet digital PCR (ddPCR) [19], detecting 14.9 ± 6% cells with HIV provirus on day 10, on average (N = 4). HIV RNA was detectable in both models at the time of RNA-Seq analysis, and increased following treatments that are used to reactivate HIV out of latency [19, 21]. In addition, a small percentage of cells generated by each of the models, without any reactivation stimuli, is p24+ (average 1.5 ± 1.2 percent and 1.8 ± 1.3 percent for bystander and cultured T_CM_ models, respectively, N = 4). A diagram in **Fig 1**shows a side-by-side comparison of the key events during the establishment of each model.

To identify genes dysregulated in latency, both T_CM_ and bystander models were compared to “mock-infected” cells cultured in parallel. These cells underwent the same exposure to activation stimuli, spin-infection, crowding and ART treatment (in case of cultured T_CM_), or exposure to proliferating cells (in case on bystander model) as their infected counterparts. This design allowed us to control for exposure of cells to cytokines and chemokines in the co-culture. Data from the cultured T_CM_ model was analyzed previously [21] identifying a total of 826 differentially expressed genes between mock-infected cells and the cells from the model of HIV latency. The bystander model was analyzed as part of the present study identifying 618 differentially expressed genes. To test the effect of the exposure to the virus on gene expression, gene expression signatures of latently infected cells were compared with a list of interferon stimulated genes with known antiviral properties [22]. This analysis identified 11 interferon stimulated genes (out of 826 total genes) for the cultured T_CM_ model and was described previously [21]. In the present study, bystander model was assessed in a similar manner, and only one interferon stimulated gene (*APOBEC3H*) was differentially expressed out of 618 total genes. We also found two upregulated DNA sensors [23], NLR family pyrin domain containing 3 (*NLRP3*) and pyrin and HIN domain family member 1 (*PYHIN1*), in cultured T_CM_ model only. Overall, these evaluations suggest that exposure to virus had minimal effect on gene expression at the time of the assay, and it may be more pronounced in the cultured T_CM_ compared to the bystander model.

### Identification of lncRNAs dysregulated in the models of HIV latency

**Fig 2:**
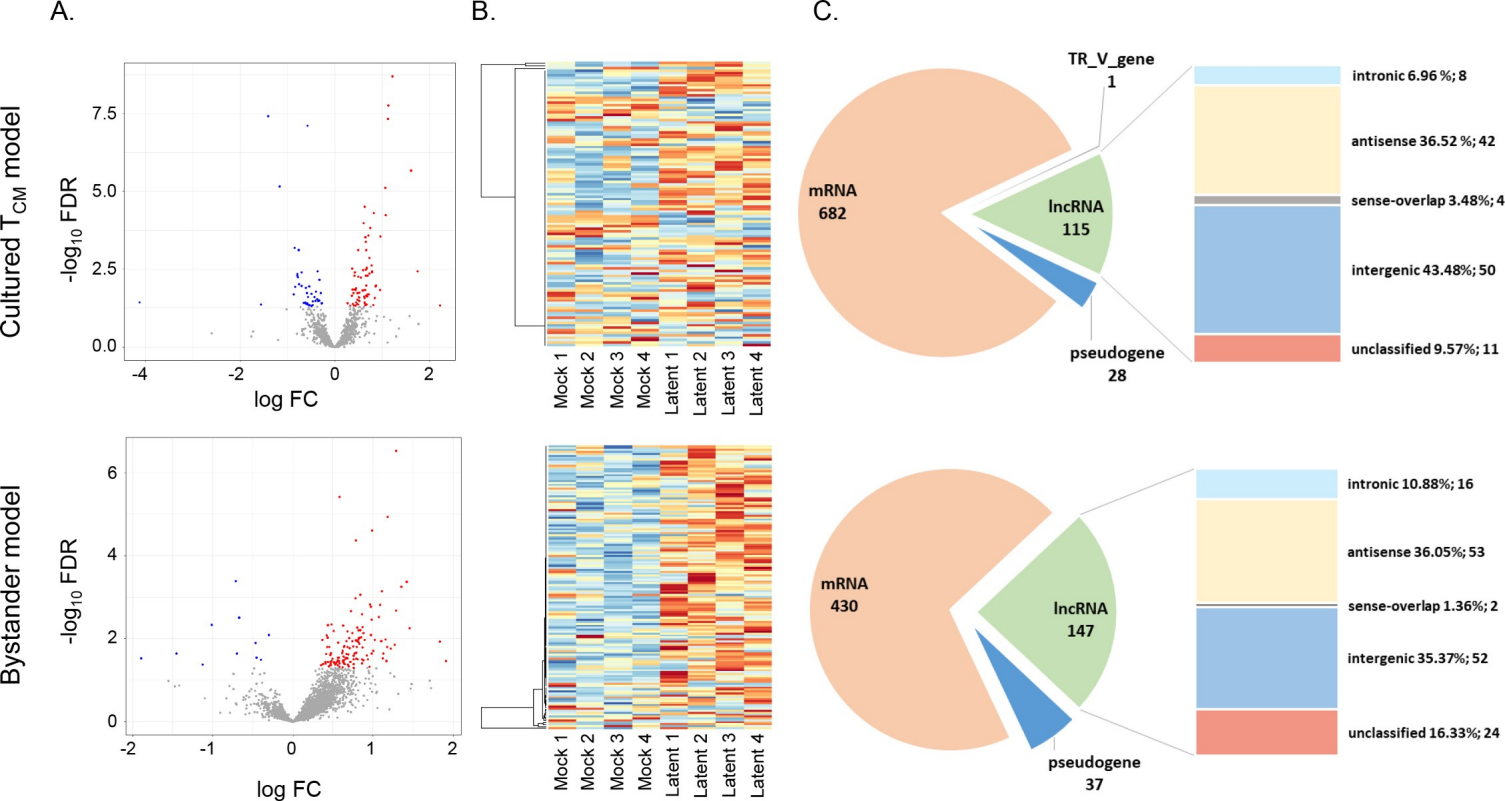
Overview of differentially expressed lncRNAs in the cultured T_CM_ and the bystander models of HIV latency. (A) Volcano plots showing all lncRNAs (grey dots). Significantly up-regulated lncRNAs (red dots) and down-regulated lncRNAs (blue dots) are highlighted for both latency models. (B) Heatmaps of differentially expressed lncRNAs. (C) Number of differentially expressed mRNAs, pseudogenes, TR_V genes, and lncRNAs, and percentages of lncRNAs of different types out of the total dysregulated lncRNAs.

Identified differentially expressed genes were annotated as protein coding or non-coding based on their types (e.g. intergenic, antisense, etc.), in both datasets (**Tables 1 and S1).** Differentially expressed lncRNAs represented 115 (13.9%) and 147 (23.8%) of all differentially expressed genes in the cultured T_CM_ and the bystander models, respectively. The majority of dysregulated lncRNA was up-regulated in both models and originated from intergenic or antisense classes, consistent with genome distribution of different lncRNA types (**Fig 2**). Intronic lncRNAs were over-represented among lncRNAs dysregulated in latency (cultured T_CM_ model: OR = 2.36, *p* = 0.02; bystander model: OR = 3.85, *p*<0.01). Differentially expressed genes and lncRNAs in each of the models of HIV latency were then compared, identifying 92 protein coding genes and 10 lncRNAs that were dysregulated in both models (**S1 Fig**). All dysregulated lncRNA were consistently either up- or down-regulated in both models (**Table 2**).

**Table 1:** Overview of differentially expressed protein coding genes and lncRNAs.

	Cultured T_CM_ model	Bystander model
	**Differential expression (gene level)**	
mRNA		
Up	456	310
Down	226	124
lncRNA		
Up	75	136
Down	40	11
Pseudogene		
Up	19	33
Down	9	4
TR_V gene		
Up	1	0
Down	0	0
Total	826	618
	**Differential expression (lncRNA types)**	
Antisense		
Up	32	48
Down	10	5
LincRNA		
Up	31	49
Down	19	3
Intronic		
Up	4	15
Down	4	1
Sense-overlapping		
Up	1	2
Down	3	0
Unclassified (TEC and proctranscript)		
Up	7	22
Down	4	2
Total	115	147

**Table 2:** LncRNAs dysregulated in common between the cultured T_CM_ and the bystander model.

			Cultured T_CM_ model			Bystander model		
ENSEMBL ID	Gene Symbol	lncRNA type	logFC	FC	FDR	logFC	FC	FDR
ENSG00000173727	*CMB9-22P13*.*1*	lincRNA	0.86	1.81	0.0115	0.52	1.43	0.0368
ENSG00000232160	*RAP2C-AS1*	antisense	0.44	1.35	0.0465	0.72	1.65	0.0021
ENSG00000245532	*NEAT1*	lincRNA	0.50	1.41	0.0438	0.37	1.30	0.0413
ENSG00000246526	*RP11-539L10*.*2*	lincRNA	-0.66	0.63	0.0377	-0.67	0.63	0.0031
ENSG00000249859	*PVT1*	lincRNA	0.37	1.29	0.0030	0.58	1.49	0.0000
ENSG00000253878	*RP11-347C18*.*3*	sense_intronic	0.73	1.66	0.0014	1.09	2.12	0.0015
ENSG00000266208	*CTD-2267D19*.*3*	antisense	0.41	1.32	0.0470	1.15	2.22	0.0105
ENSG00000267702	*RP11-53B2*.*2*	sense_intronic	0.51	1.42	0.0253	0.56	1.48	0.0125
ENSG00000271122	*RP11-379H18*.*1*	antisense	0.38	1.30	0.0145	0.37	1.29	0.0107

*logFC*, log_2_-transformed fold change of counts per million in the RNA-Seq data; *FC*, fold change; *FDR*, false discovery rate-corrected *p*-value.

### Validation of expression of lncRNA by ddPCR

Though an overlapping set of dysregulated lncRNAs was identified using RNA-Seq, the difference in expression of lncRNAs between the model of latency and mock-infected cells was frequently less than the effect size of the RNA-Seq experiment (effect size ≈1.5 with N = 4 biological replicates). Small differences in expression were not unexpected because each model of HIV latency is comprised of the mixture of uninfected and latently infected cells, with latently infected cells ranging from 8.8% to 44.3% [19, 21] (defined as percentage of cells with HIV provirus). Therefore, we aimed to use a method independent of RNA-Seq to validate expression of a set of overlapping lncRNAs. DdPCR was chosen for this purpose, because it is the most sensitive technique for detection of small differences in gene expression. Four lncRNAs, *PVT1* oncogene (Ensembl ID ENSG00000249859), *RP11-347C18*.*3* (Ensembl ID ENSG00000253878), *RP11-539L10*.*2* (Ensembl ID ENSG00000246526) and *NEAT1* (Ensembl ID ENSG00000245532), were selected for validation. Same samples that were sequenced, and three additional sample pairs for the bystander model were analyzed. Up-regulation of *PVT1* and *RP11-347C18*.*3* was confirmed both in the cultured T_CM_ and the bystander models (cultured T_CM_ model: *PVT1* average fold change 1.8, *p* = 0.02; *RP11-347C18*.*3* average fold change 2.95, *p* = 0.01; bystander model: *PVT1* average fold change 1.33, *p* = 0.02; *RP11-347C18*.*3* average fold change 2.12, *p* = 0.0002) (**S2 Fig**). Down-regulation of *RP11-539L10*.*2* was confirmed in the bystander model (average fold change -1.28, *p* = 0.046) (**S2 Fig**). Up-regulation of *NEAT1* was not confirmed in either of the models, neither with the assay that detects both short and long isoforms (N1+N2), nor the long isoform-specific assay (N2) (**S2 Fig**).

Neither of the RNA-Seq datasets contained the Ensembl identifier for lncRNA *NRON* (ENSG00000253079), possibly because it was not present in the reference files used for mapping and counting RNA-Seq reads. Because *NRON* represents the only lncRNA whose function in maintaining HIV in latent state has been demonstrated in a primary T-cell model system and cell from HIV-infected individuals [8], we measured the expression of *NRON* using ddPCR. Despite the previous report of a relatively high expression of this lncRNA in primary CD4+ T-cells [8], we observed relatively low expression, two orders of magnitude lower than of the other lncRNAs measured. For the cultured T_CM_ model, there was sufficient amount of RNA to measure *NRON* from only three out of four sequenced sample pairs. In these three donors, *NRON* was strongly and significantly up-regulated (average fold change 4.2, *p* = 0.003) in the model of latency compared to mock-infected cells (**S2 Fig**). Though in some cases *NRON* was up-regulated in the bystander model, up-regulation was modest (average fold change 1.2 for four donors and 1.1 for 7 donors) and not consistent among the seven replicate experiments (**S2 Fig**). Thus, though up-regulation of *NRON* in latency may be used as validation of our approach of identifying lncRNAs that function in latency control, the discrepant results for *NRON* between the two models are consistent with the idea that route of latency establishment and cell composition may contribute to the mechanisms by which latency is regulated.

### LncRNAs dysregulated in the models of HIV latency have higher expression in memory compared to naïve T-cells

**Fig 3:**
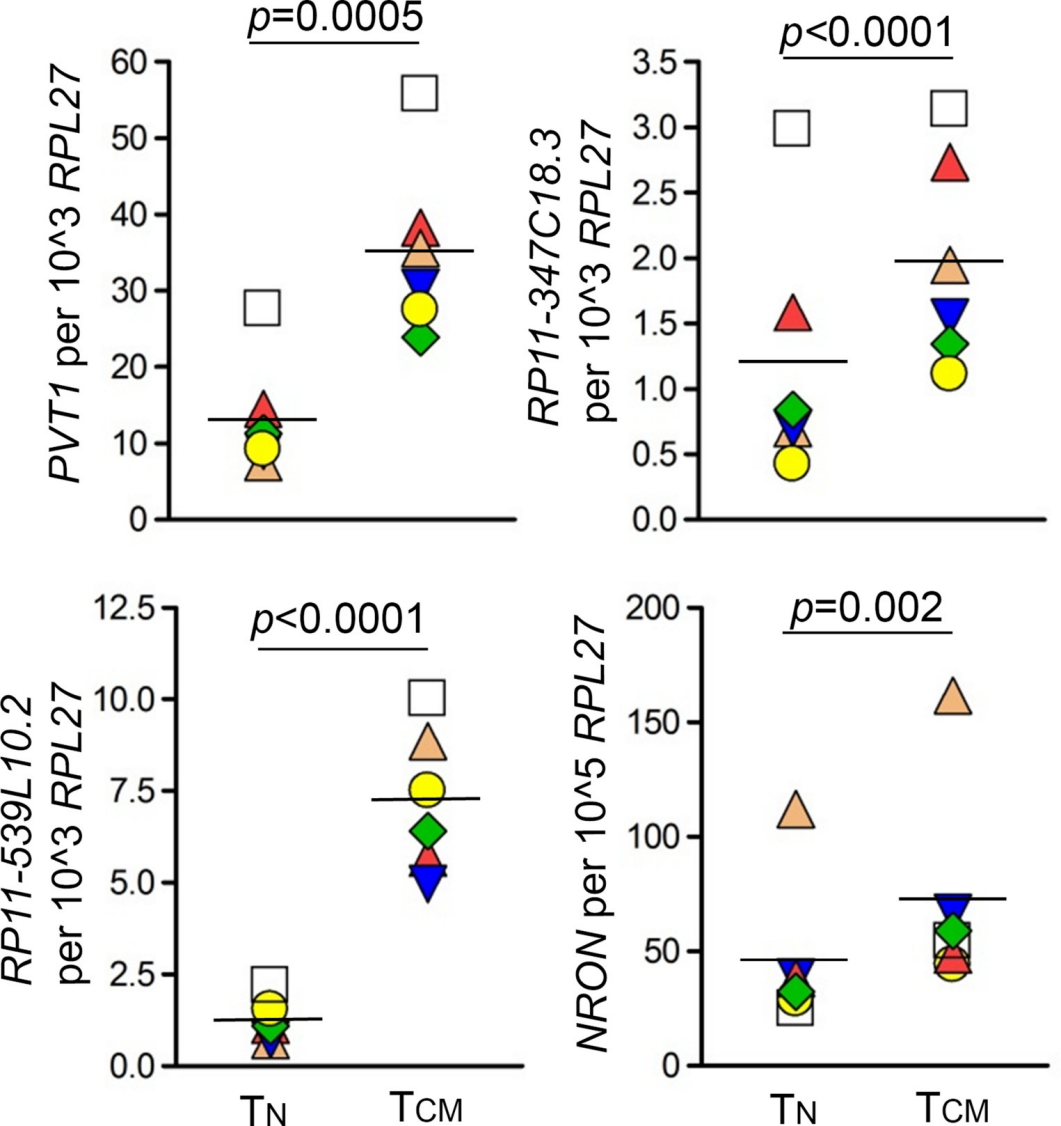
Comparison of lncRNA expression in T_N_ and T_CM_ T-cells. Expression of *PVT1*, *RP11-347C18*.*3*, *RP11-539L10*.*2* and *NRON* was measured by ddPCR and normalized to expression of the housekeeping gene *RPL27*. Six independent experiments were performed using uninfected CD4+ T-cells. Significance was determined using a paired two-sided *t*-test for log_2_ transformed data. Data is presented as individual data points (copy numbers normalized to *RPL27*) symbol-coded by donor, mean of all values is shown. Samples were generated using blood from donors who were different from those who participated in the study that used models of HIV latency. *T*_*N*_, naïve CD4+ T-cells; *T*_*CM*_, central memory CD4+ T-cells.

Memory cells are considered to be the major reservoir of latent HIV provirus [24–28]. If a host factor functions to promote HIV latency, its expression would likely be higher in memory than in T_N_ cells. Indeed, an HIV repressor, Blimp-1 (gene symbol *PRDM1*) was expressed at significantly higher levels in T_CM_ than in T_N_ cells [29]. Therefore, to further assess whether identified lncRNAs likely function in latency control, we measured the expression of *PVT1*, *RP11-347C18*.*3*, *RP11-539L10*.*2* and *NRON* in T_N_ and memory cell subsets, including T_CM_ and T_EM_ cells. Uninfected cells sorted into major phenotypic subsets were used for this experiment. While *PVT1* and *RP11-347C18*.*3* were up-regulated and *RP11-539L10*.*2* was down-regulated in latency, expression of all three lncRNAs was significantly higher in T_CM_ and T_EM_ compared to T_N_ cells (**Figs 3 and S3**). Specifically, *PVT1* expression was on average 3-fold higher in T_CM_ than T_N_ cells (N = 6, *p* = 0.0005); and on average 2.8-fold higher in T_EM_ than T_N_ cells (N = 4, *p*<0.0001). *RP11-347C18*.*3* expression was on average 2-fold higher in T_CM_ (N = 6, *p*<0.0001) or T_EM_ (N = 4, *p*<0.0001) than T_N_ cells. *RP11-539L10*.*2* expression was on average 6.6-fold higher in T_CM_ than T_N_ cells (N = 6, *p*<0.0001); and on average 20-fold higher in T_EM_ than T_N_ cells (N = 4, *p*<0.0001). Consistent with its repressive function for HIV, *NRON* also had higher expression in T_CM_ and T_EM_ compared to T_N_ cells (**Figs 3 and S3)** (average 1.7-fold higher in T_CM_ than T_N_ cells (N = 6, *p* = 0.002); average 4.1-fold higher in T_EM_ than T_N_ cells (N = 3, *p<*0.001)). Because *PVT1* and *RP11-347C18*.*3* were up-regulated in latency and in memory cells, their predicted function would be repression of HIV expression. In contrast, because *RP11-539L10*.*2* was down-regulated in latency and had higher expression in memory cells, it would be predicted to promote HIV expression.

### Identification of cellular pathways associated with lncRNAs dysregulated in the models of HIV latency

**Fig 4:**
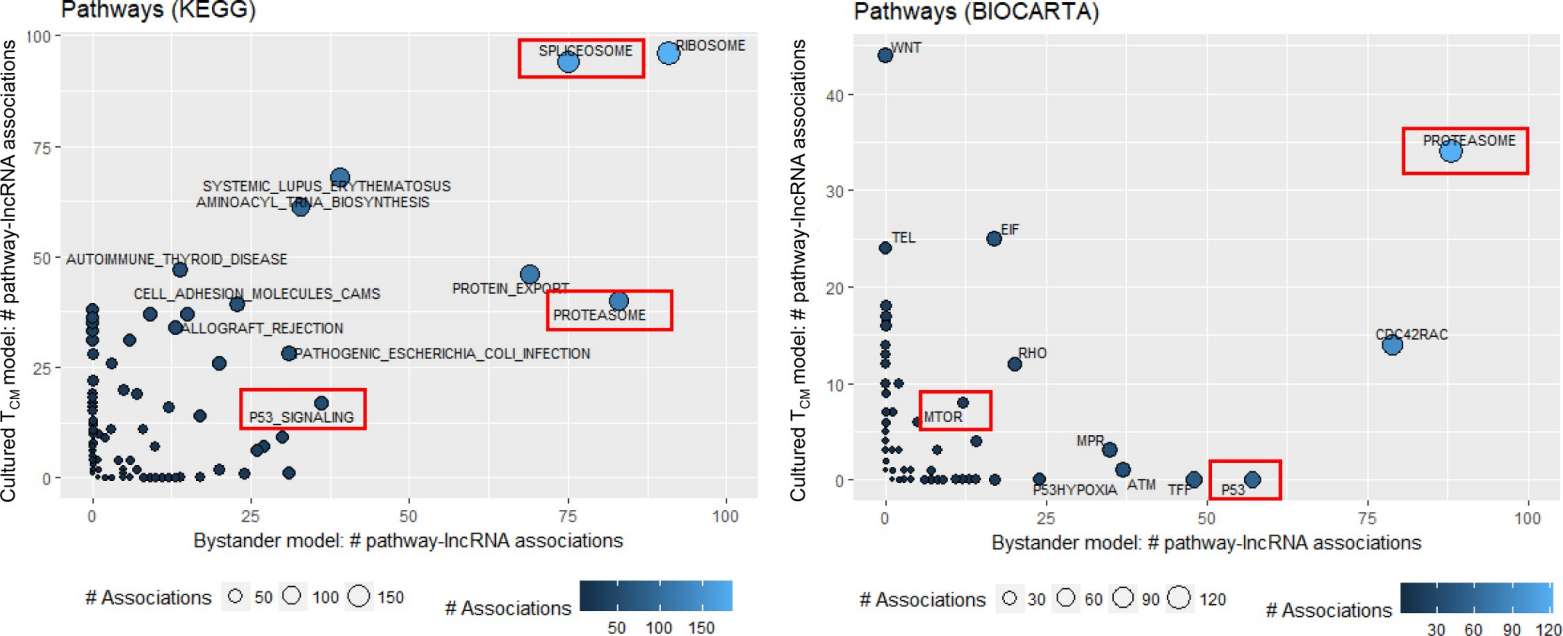
Pathways associated with lncRNA dysregulated in latency, identified by the guilt-by-association (GBA) analysis. Expression of lncRNAs dysregulated in latency (EdgeR’s FDR corrected *p*-value<0.05) was correlated with expression of all detected mRNAs. A ranked mRNA gene list was constructed for each lncRNA based on their correlation coefficients and used as input for gene set enrichment analysis (GSEA) with KEGG and Biocarta pathway databases. A lncRNA was considered significantly associated to a pathway when the GSEA FDR corrected *p*-value was less than 0.05. Number of lncRNA pathway associations is shown on the X-axis for the bystander model and Y-axis for cultured T_CM_ model, color-coded with blue shades and indicated by the size of the circle (lighter blue color and larger circle size correspond to the greater number of associations). Ribosome and spliceosome pathways have the most associations with dysregulated lncRNAs in both models. Pathways that line up along the X axis were identified for the bystander model dataset only; while pathways that line up along the Y axis were identified for the cultured T_CM_ model only. Pathways relevant to HIV latency are highlighted by red boxes.

We further inferred function of dysregulated lncRNAs in HIV latency using GBA analysis, which determines correlations between lncRNA and mRNA expression in combination with enrichment strategies to identify cellular pathways associated with lncRNAs. Among the identified pathways, some were found in association with dysregulated lncRNA for both the cultured T_CM_ and the bystander models (**S4 and S5 Figs)**. Specifically, Spliceosome, Ribosome, Proteasome, and Protein export pathways from the Kyoto Encyclopedia of Genes and Genomes (KEGG), and Proteasome pathway from Biocarta database were associated with multiple dysregulated lncRNAs in both models (**Fig 4**). Of these, proteasome function has been previously implicated in maintaining HIV in latent state, and proteasome inhibitors were proposed as LRAs [30, 31]. Among other pathways with proposed roles in HIV latent reservoir establishment or maintenance, were p53 signaling [21] and mammalian target of rapamycin (MTOR) [32]. KEGG p53 signaling pathway had many lncRNA associations in both models, while lncRNA associations with Biocarta p53 pathway was only found for the bystander model (**Fig 4)**. Fewer lncRNAs were associated to Biocarta MTOR, but for both models of HIV latency (**Fig 4)**. Proper splicing is important for HIV protein production toward viral replication, but also toward antigen presentation to the immune system for killing of infected cells. Therefore, we and others propose that regulation of the spliceosome can critically regulate viral latency through multiple mechanisms [33, 34]. Through down-regulation of singly-spliced and unspliced messages, the virus can remain in a non-replicative state while being invisible to the immune system. Favoring certain doubly-spliced messages, specifically those encoded by Nef, the virus can also down-regulate molecules required for antigen presentation by the infected cell. Thus, it is plausible to hypothesize that spliceosome pathway may also have relevance to regulation of HIV latency. In addition, splicing factors that contain serine/arginine (SR) domains are well known regulators of HIV splicing [35]. Dysregulated lncRNA were found in association with several genes that encode SR protein mRNAs: *SRSF2*, *SRSF3*, *SRSF6*, *SRSF7* and *SRSF10*.

**Fig 5:**
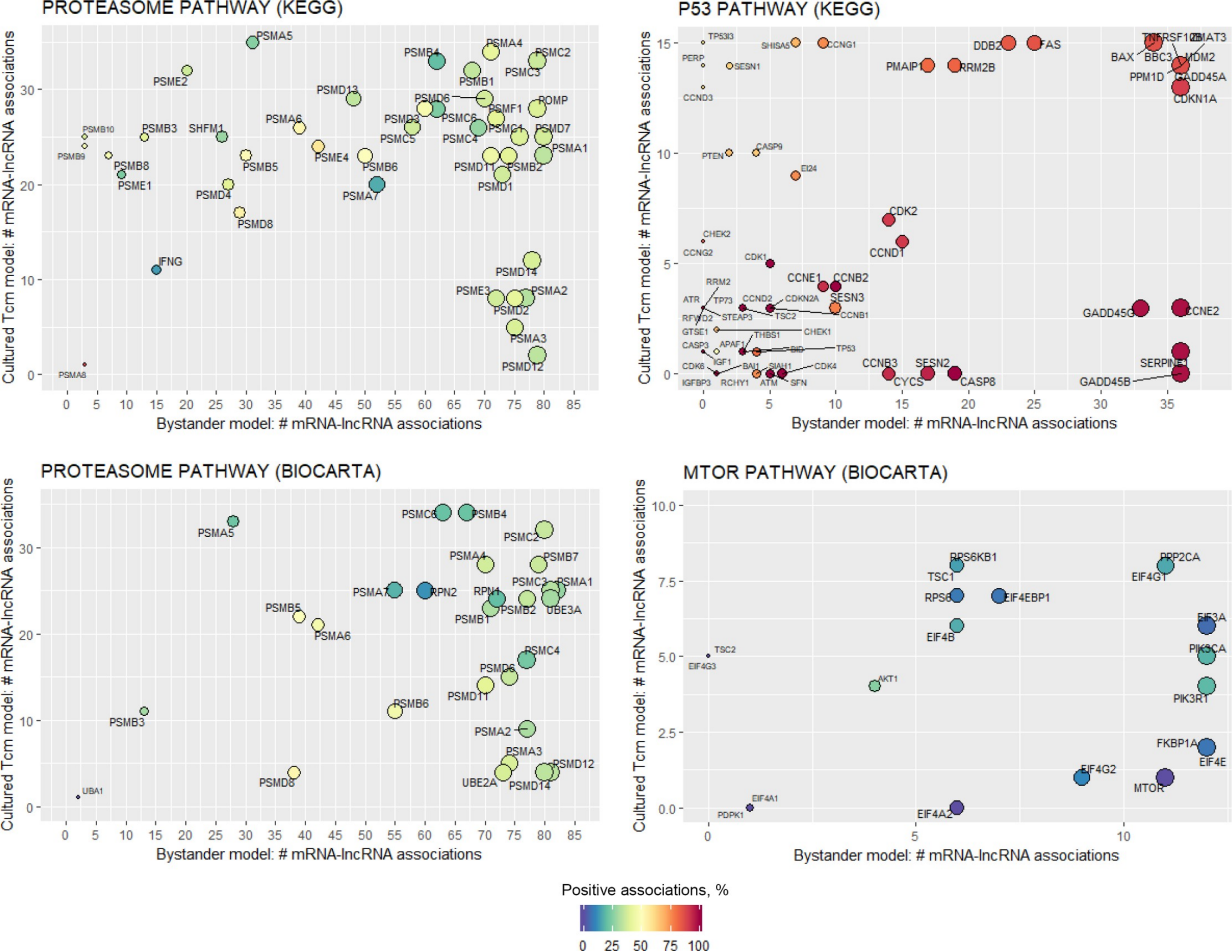
Associations between dysregulated lncRNAs and the mRNA components of pathways implicated in HIV latency. For each lncRNA that was significantly associated with a pathway of interest (red boxes in Fig 4), leading edge mRNA genes were identified that drive the pathway enrichment score (GSEA). Next, the number of lncRNAs associated to pathway-specific mRNAs were plotted (*ggplot2* v2.2.1). Number of associations between mRNA and lncRNA is shown on the X-axis for the bystander model and Y-axis for cultured T_CM_ model, and indicated by the size of the circle (larger circle size corresponds to the greater number of associations). Color shows the percentage of positively correlated mRNA/lncRNA pairs (dark red is 100% positive correlations; dark blue–no positive correlations; yellow–equal number of positive and negative correlations).

Next, we explored the associations between lncRNA expression and expression of individual members (mRNAs) of pathways implicated in HIV latency. Expression of the individual genes of the KEGG p53 signaling pathway positively correlated with expression of dysregulated lncRNAs, while Biocarta MTOR showed mostly negative associations (**Fig 5**). Both KEGG and Biocarta proteasome pathway exhibited mostly negative associations (**Fig 5**). Many mRNAs with highest number of associations to dysregulated lncRNAs were shared for both proteasome and MTOR pathways. In contrast, p53 pathway had a cluster of mRNAs commonly associated with dysregulated lncRNAs for both the cultured T_CM_ and the bystander models (e.g. top right on the top panel in **Fig 5**), while other mRNAs were model-specific (e.g. a cluster of genes specific for the bystander model of HIV latency at the bottom right on the top panel in **Fig 5**). Overall, these data are consistent with a possibility that these pathways are regulated via multiple lncRNAs, and that control by lncRNA has both shared and unique components of the same pathway.

### *PVT1* and *RP11-347C18*.*3* are associated with pathways implicated in regulation of HIV latency

**Fig 6:**
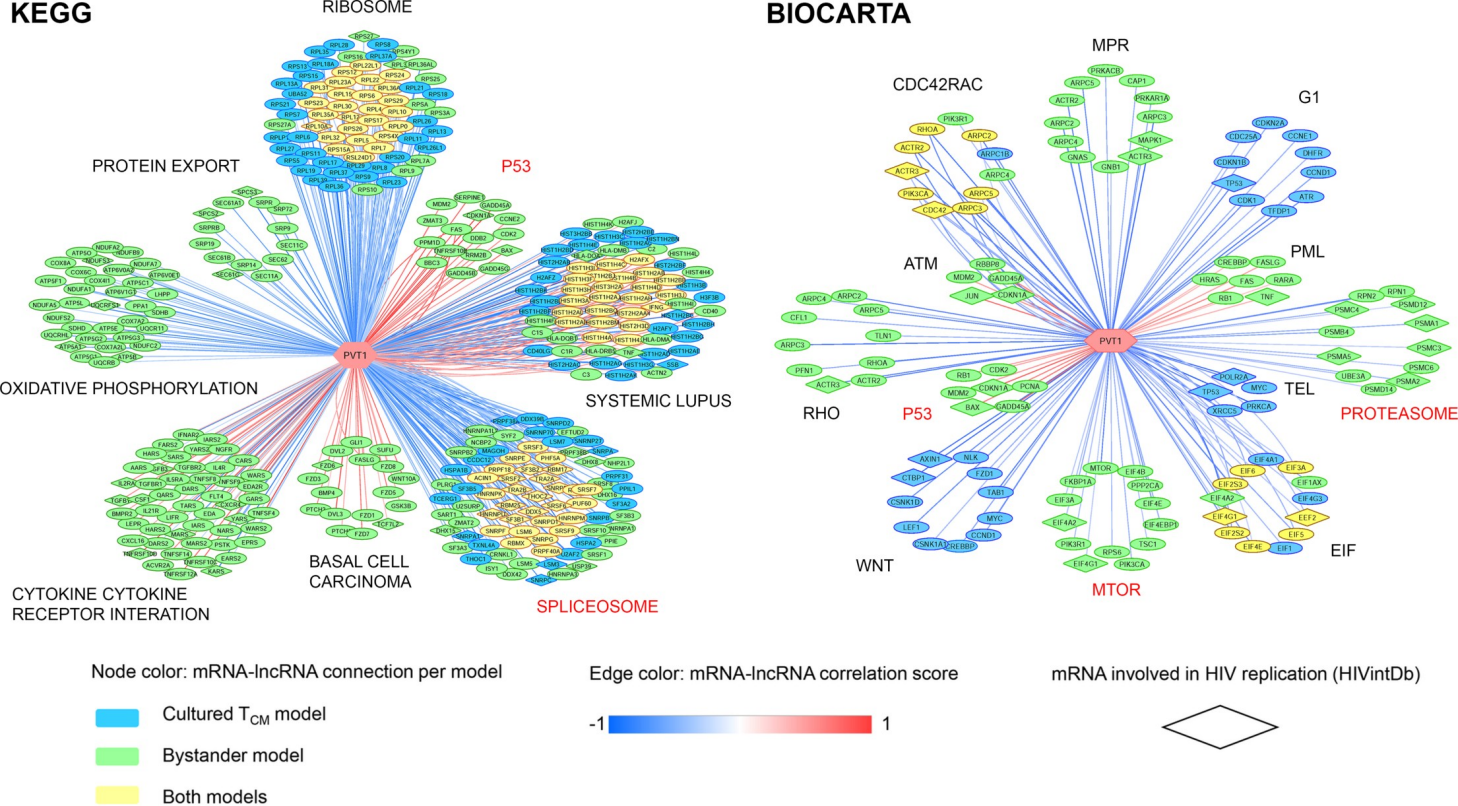
Associations between *PVT1* and mRNA components of identified associated KEGG and Biocarta pathways. Output of the guilt-by-association analysis was filtered for *PVT1*-specific pathway associations and corresponding leading edge mRNAs (GSEA FDR < 0.05). Next, a matrix with *PVT1*-specific information on pathway association, HIV latency model, enrichment score and leading edge mRNAs served as input for Cytoscape visualization (v3.4.0). Associations between *PVT1* and mRNAs in all pathways (FDR corrected *p*<0.05) are shown. Nodes represent individual mRNAs associated with *PVT1* and are color coded by model of HIV latency: *blue*, cultured T_CM_ model, *green*, bystander model, and *yellow*, common association for both models. Nodes in a shape of a rhombus indicate mRNAs involved in HIV replication (annotated in the NCBI HIV-1 Interaction Database, *HIVintDb*). The color of the lines connecting *PVT1* to mRNAs indicate correlation (*red*, positive; *blue*, negative). Names of pathways implicated in HIV latency are indicated in *red*.

We further focused our attention on lncRNAs that were dysregulated in both models of latency and validated by ddPCR, namely *PVT1*, *RP11-347C18*.*3* and *RP11-539L10*.*2*. We aimed to determine whether these lncRNAs were specifically associated with pathways implicated in regulation of HIV expression. Of these, *PVT1* exhibited the most associations (**Fig 6**), including both KEGG and Biocarta p53 signaling pathway, Biocarta MTOR and proteasome pathways, and KEGG spliceosome pathway. Most of these associations were found for the bystander model and not the cultured T_CM_ model; however, spliceosome, and a number of SR splicing factors in particular associated with *PVT1* for both models (**Fig 6**). *RP11-347C18*.*3* was associated with p53 signaling, both for KEGG and Biocarta pathways, and with the KEGG spliceosome pathway (**S2 Table**). *RP11-539L10*.*2* was not associated with any annotated pathways implicated in HIV latency (**S2 Table**). The role of *RP11-539L10*.*2* lncRNAs in regulation of HIV latency cannot be excluded based on these results, because it may function via control of a single critical host gene or its protein product, by directly targeting HIV, or via a pathway that is not annotated in the KEGG or Biocarta database. However, roles for *PVT1* and *RP11-347C18*.*3* are more likely based on identification of multiple HIV latency-related pathways associated with these lncRNAs.

### Treatment with SAHA and RMD results in down-regulation of lncRNAs dysregulated in the models of HIV latency

**Fig 7:**
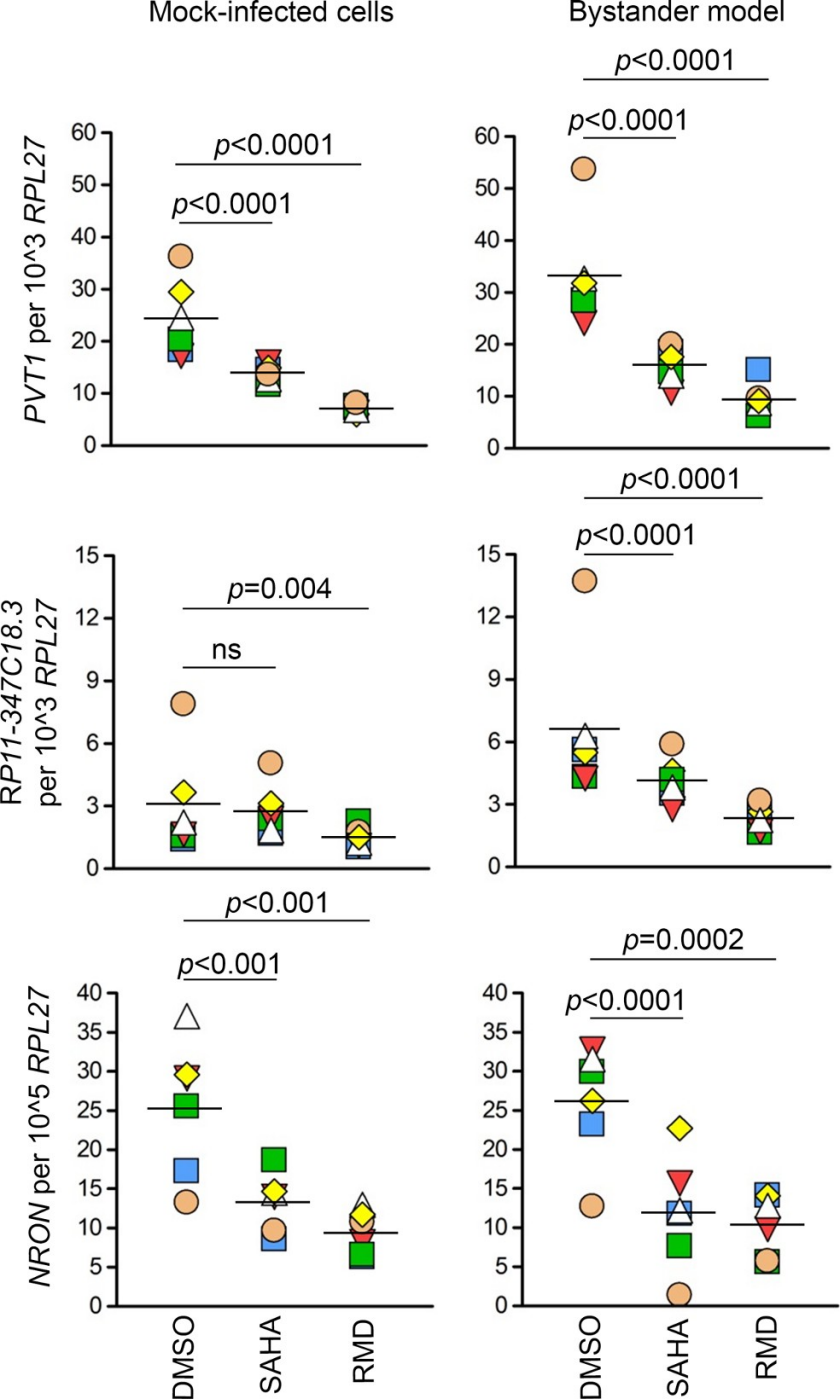
Down-regulation of *PVT1*, *RP11-347C18*.*3* and *NRON* by SAHA and RMD. Mock-infected cells and the bystander model of HIV latency were treated with SAHA (1 μM), RMD (15 nM) or their solvent DMSO for 24 hours. Expression of lncRNAs was measured by ddPCR and normalized to expression of the housekeeping gene *RPL27*. Six replicate experiments were performed. Significance was determined by implementing repeated measures analysis of variance (RM ANOVA) with library *nlme* in R using log_2_ transformed data. Data is presented as individual data points (copy numbers normalized to *RPL27*) symbol-coded by donor, mean of all values is shown. *DMSO*, dimethyl sulfoxide; *SAHA*, suberoylanilide hydroxamic acid; *RMD*, Romidepsin; *ns*, not significant (*p*-value > 0.05).

Finally, we have assessed how treatment with clinically tested LRAs, histone deacetylase inhibitors (HDACi) SAHA and RMD, affects expression of dysregulated lncRNAs. Samples from four to six replicates from the bystander model were used for this experiment. *PVT1* and *NRON* were down-regulated by SAHA and RMD in the bystander model and mock-infected cells (*PVT1*: SAHA mock average fold-change -1.78, RMD mock average fold change -3.48, SAHA model of latency average fold change -2.1, RMD model of latency average fold change -2.8, *p*<0.0001; *NRON*: SAHA mock average fold-change -1.9, RMD mock average fold change -2.8, SAHA model of latency average fold change -3.5, RMD model of latency average fold change -2.8, *p*<0.001) (**Fig 7**). *RP11-347C18*.*3* was down-regulated in all conditions except by SAHA in mock-infected cells (RMD mock average fold change -2.0, *p* = 0.004; SAHA model of latency average fold change -1.56, RMD model of latency average fold change -2.7, *p*<0.0001) (**Fig 7**). *RP11-539L10*.*2* was down-regulated in all conditions except by RMD in the bystander model of HIV latency (SAHA mock average fold-change -3.81, RMD mock average fold change -3.43, *p*<0.0001; SAHA model of latency average fold change -2.25, *p* = 0.002) (**S6 Fig**). Overall, these results demonstrate that the effects of SAHA and RMD on expression of selected lncRNAs appear to be independent from HIV infection. As the primary mechanism of action of HDACi is chromatin decondensation leading to induction of gene transcription, down-regulation of the tested lncRNAs most likely represents a secondary mechanism of action of these compounds.

## Discussion

The present study aimed to prioritize lncRNAs to be tested as targets for HIV latency reversal. We used several complementary approaches, including the identification of dysregulated lncRNAs in primary T-cell models of HIV latency (Figs 2 and S1 and S2and Tables 1, 2 and S1), GBA analysis (Figs 4–6 and S4 and S5 and S2 Table), evaluation of lncRNA expression in major CD4+ T-cell maturation subsets (Figs 3 and S3) and in CD4+ T-cells treated with clinically tested LRAs (Figs 7 and S6.

Among lncRNAs dysregulated in the models of HIV latency, 10 were in common between the two models (**Table 2**). In addition, 105 unique lncRNAs were identified for the cultured T_CM_ model, and 137 for the bystander model (**S1 Fig**). Same protocols were followed to generate and analyze RNA-Seq data for both models (please refer to Materials and Methods); thus, discrepancies in the result caused by differences in experimental and analytical procedures are expected to be minimal. Therefore, it is likely that the observed differences in the identified dysregulated lncRNAs were due to biological differences of the two models. Specifically, T-cell composition and route of latency establishment may contribute to differential dysregulation of lncRNAs in latency. One observation in favor of this conclusion is that *NRON* was strongly up-regulated in the cultured T_CM_ model that establishes latency in activated cells returning to quiescence, but not in the bystander model that uses direct infection of resting cells (**S2 Fig**). A prior study that demonstrated the role of *NRON* in latency maintenance [8] used a model with a similar route for latency establishment as our cultured T_CM_ model.

We further focused our evaluation of possible functions of the four lncRNAs that were dysregulated in both models of latency and had higher expression levels among the ten lncRNA identified, namely *PVT1*, *RP347C18*.*3*, *RP539L10*.*2* and *NEAT1*. Of these, *NEAT1* differential expression was not validated by ddPCR in either model (**S2 Fig**). The prior studies demonstrating the role of *NEAT1* in HIV RNA nuclear retention during productive HIV replication was performed using cell lines [6, 7]. Therefore, it is possible that *NEAT1* may not function in the same manner in latently infected primary CD4+ T-cells. Because the effect size with the number of samples (N = 4) available in our RNA-Seq experiment was ≈1.5, and the observed difference for *NEAT1* was smaller (1.41-fold in the cultured T_CM_ model and 1.3-fold in the bystander model, **Table 2**), *NEAT1* might represent one of the false positives detected in our RNA-Seq study. The remaining three selected lncRNAs were confirmed by ddPCR and evaluated in CD4+ T-cells of naïve and memory phenotypes. All of them exhibited higher expression in T_CM_ and T_EM_ cells (**Figs 3 and S3**), which represent the major component of the latent reservoir. *PRDM1*, a known HIV repressor whose function was evaluated in primary CD4+ T-cells, exhibited a similar pattern of expression in T_N_ and T_CM_ subsets [29]. LncRNA *NRON*, whose repressive function for HIV expression was previously demonstrated in primary CD4+ T-cells [8], also had higher expression in T_CM_ and T_EM_ compared to T_N_ cells (**Figs 3 and S3**). Therefore, our observation of elevated expression of selected lncRNAs in memory cells is consistent with their possible function in regulation of HIV expression in latency.

GBA analysis was used to hone into potential functions of dysregulated lncRNAs in HIV latency control by determining whether they are associated to known pathways implicated in establishment or maintenance of HIV latency. GBA analysis identified proteasome, spliceosome, p53 signaling and MTOR among pathways associated with dysregulated lncRNAs (**Figs 4 and S4 and S5**). Most of the lncRNAs had negative associations with mRNA members of these pathways (**Fig 5**), consistent with a possibility of inhibitory regulation of gene expression of these pathways by lncRNA. Some of the mechanisms by which this regulation may occur include transcriptional interference, promoter inactivation by binding transcription factors, cytoplasmic retention of transcription factors, and epigenetic silencing [13, 36]. In contrast, the majority of associations in the p53 signaling pathway were positive (**Fig 5**). Such positive regulation may as well occur through chromatin remodeling and interactions with and recruitment of transcriptional activators [13]. In addition, lncRNAs may function by increasing stability of the mRNAs [37]. Negative [38] as well as positive [39] feedback loops have been reported between lncRNAs and proteins; however, independent studies of interactions between the identified lncRNAs and the members of associated pathways at the protein level would be required to determine whether this may be the case for regulation of HIV latency.

Of the three dysregulated lncRNAs that were validated by ddPCR, *PVT1* was found in association with multiple pathways implicated in control of HIV latency (**Fig 6**). The common pathway identified for the cultured T_CM_ and the bystander models associated with *PVT1* was spliceosome (**Fig 6**). Remarkably, *PVT1* was associated with a number of mRNAs encoding SR proteins, in common between the two models (**Fig 6**). Of these, *SRSF2*, *SRSF6*, and *SRSF10* have been specifically linked to HIV splicing [40–43]. *PVT1* was associated with members of p53 signaling, MTOR and proteasome pathway in the case of the bystander model only (**Fig 6**), consistent with the idea that pathways that regulate latency may be, at least in part, dependent on the model. *PVT1* was found in association with p53 signaling pathway for both KEGG and Biocarta databases (**Fig 6**). The role of p53 signaling in HIV establishment and maintenance was demonstrated previously [21]. One protein member of this pathway, MDM2 Proto Oncogene (*MDM2*), was stabilized by *PVT1* via its interaction with Enhancer Of Zeste 2 Polycomb Repressive Complex 2 Subunit (*EZH2*) in the setting of hepatocellular carcinoma [44]. Though the precise mechanism of regulation of p53 signaling pathway by *PVT1* in different cell types needs further investigation, the positive regulation of it components was consistent in our study and the study by Guo and colleagues [44]. In addition, *EZH2* itself contributes to regulation of HIV latency [45]. *PVT1* interacted with and stabilized the *EZH2* protein product [44], which could represent a potential mechanism of latency control by *PVT1*. In addition, *PVT1* was shown to act as a sponge for multiple miRNAs [46–51], suggesting the possibility for positive regulation of multiple mRNAs in the p53 signaling pathway that represent miRNA targets. Based on these results, we speculate that *PVT1* may function in HIV latency control via modulating mRNA components of p53 signaling pathway and splicing machinery.

Intriguingly, identified associations of *PVT1* to cellular pathways demonstrate possible links between inferred mechanisms of regulation of gene expression by *PVT1* in HIV latency and other diseases, such as basal cell carcinoma and systemic lupus erythematosus (**Fig 6**). We speculate that a subset of dysregulated genes that may be regulated by *PVT1* is shared between these conditions. For example, *TCF7L2* (also known as TCF-4) from the basal cell carcinoma pathway plays a role in HIV latency maintenance [52] and was up-regulated in the bystander model in our study (**S1 Table**). For the systemic lupus erythematosus pathway, the majority of genes linked to *PVT1* are histone-coding genes. Epigenetic alterations, histone modifications in particular, largely contribute to gene expression dysregulation in systemic lupus erythematosus [53]. A number of histone-coding genes were dysregulated at the RNA level in systemic lupus erythematosus [54] and the cultured T_CM_ model in our study (**S1 Table**).

Lastly, our study investigated the effects of clinically tested LRAs, SAHA and RMD, on expression of lncRNAs dysregulated in latency, *PVT1*, RP11-347C18.3, *RP11-539L10*.*2*, and *NRON*. SAHA and RMD are HDACi, whose primary mechanism of action is histone hyperacetylation which results in chromatin decondensation and elevated gene expression. However, multiple studies demonstrated that HDACi cause gene down-regulation [55–57], which likely represents secondary mechanisms of action of these compounds. Our prior investigation of these secondary effects on protein coding genes suggested presence of both stimulatory and inhibitory effects of HDACi with respect to HIV reactivation [19, 58]. All tested lncRNAs, *PVT1*, *RP11-347C18*.*3*, *RP11-539L10*.*2*, and *NRON* were down-regulated by SAHA and RMD, independent of the presence of latent infection (Figs 7 and S6). When HIV is present, down-regulation of *PVT1*, *RP11-347C18*.*3*, and *NRON*, which were up-regulated in latency, may represent stimulatory effects. Further down-regulation of *RP11-539L10*.*2* by SAHA, beyond levels of down-regulation observed in latency compared to mock-infected cells, may represent an inhibitory effect. Thus, both stimulatory and inhibitory effects of HDACi are likely present among non-coding genes. The results from this study provide basis for hypothesis building to identify candidate lncRNAs whose experimental down-regulation (such as via knockdowns) may be synergistic with HDACi treatment for latency reversal.

One limitation of the present study is the low frequency of latently infected cells in a large background of uninfected cells for both models of HIV latency (range 8.8% to 44.3% [19, 21]). This is a common problem for models that use replication-competent wild type HIV virus. Therefore, we conducted a comparison of dysregulated lncRNAs in two different well characterized models [16–19, 21, 59] to attempt to overcome the limitation introduced by high background of uninfected cells. Only lncRNAs found dysregulated in both models were analyzed in more detail. Characterization of single cell transcriptomes using the cultured T_CM_ and bystander models might be beneficial for identification of a greater number of overlapping lncRNAs and for validating the role of T-cell maturation state and prior activation in lncRNA expression signatures of latently infected cells.

The second limitation is a possibility that differential gene expression may result from the exposure of cells to cytokines and chemokines produced by cells during productive infection, or due to exposure to the virus in culture. To control for cytokine and chemokine exposure during T-cell activation, we ensured that the mock-infected cells were treated the same way as infected cells throughout the culture. We have also searched for interferon stimulated genes with known antiviral properties [22] and DNA sensors [23] among genes that were differentially expressed in the latency models compared to mock-infected cells. Some such responses were detected, predominantly in the cultured T_CM_ model. It is also possible that transcriptional profiles could be influenced by virus via interferon-independent cascades [60, 61]. While a good control for these effects might be the use of anti-virals during the stages of latency establishment, or the use of cells exposed to the aldrithiol-2 inactivated virus [62] instead of mock-infected cells, we were limited by analyzing previously published RNA-Seq datasets that did not include these controls. Nonetheless, identification of a subset of genes with known roles in HIV latency supports the potential of this experimental and analytical approach to identify genes specifically affected during HIV latency and not purely by cytokines or exposure to virus. Indeed, expression of p21 (gene symbol *CDKN1A*) was elevated in the cell line model of latency [63] and in the bystander model in the present study (**S1 Table**). Its expression negatively correlated with cell-associated HIV RNA and HIV transcriptional activity in HIV-infected virologically suppressed individuals on cART [64], and its protein product inhibited transcription from the integrated provirus via inhibition of Cyclin Dependent Kinase 9 (CDK9), a component of positive transcription elongation factor (P-TEFb) [65]. Another example is *PRDM1*, up-regulated in both models in the present study (**S1 Table**), which was previously shown to be a repressor of basal and Tat-dependent HIV transcription [29]. Moreover, expression of the only lncRNA with demonstrated role in HIV repression in primary CD4+ T-cells, *NRON* [8], was also up-regulated in the cultured T_CM_ model in the present study (**S2 Fig**). These observations are consistent with the idea that at least a subset of genes and lncRNAs found dysregulated in latency in the present study has demonstrated roles as HIV regulators. Ultimately, identified lncRNAs will need to be validated as HIV regulators using functional studies (e.g. experimental gene expression knockdowns) using relevant model systems and cells from HIV-infected individuals *ex vivo*.

In summary, the present study used gene expression profiling by RNA-Seq in two primary T-cell models of HIV latency to focus on identification of dysregulated lncRNAs. Despite differences in cell composition and route of latency establishment between the models, common lncRNA signatures associated with latency could be identified. LncRNAs dysregulated in latency had higher expression in memory cells that represent the major HIV reservoir, as compared to T_N_ cells. We further identified pathways associated with dysregulated lncRNA, including pathways previously implicated in HIV latency: proteasome, spliceosome, p53 signaling and MTOR. Of the lncRNAs that were dysregulated in common between the two models, *PVT1* had the most associations to these pathways. We have proposed several mechanisms of action for *PVT1* to regulate HIV latency, based on evidence for *PVT1* function from published literature. Identification of model-specific dysregulated lncRNAs and lncRNA-associated pathways will facilitate better understanding of the mechanisms by which latency is established and maintained in different cell types. The role of the identified lncRNAs in HIV latency and their mechanisms of action warrant further experimental exploration to determine their suitability as targets for antiviral strategies. Our study facilitates prioritization of lncRNAs to be tested in this setting.

## Materials and methods

### Study participants

Primary CD4+ T-cells from HIV seronegative volunteer blood donors were used for this study. The protocol was approved by the Institutional Review Boards of the University of California San Diego, and the VA San Diego Healthcare System and abides by the Declaration of Helsinki principles. All volunteers gave written informed consent to participate in the study.

### *In vitro* models of HIV latency

To identify lncRNAs dysregulated in HIV latency, two primary T-cell models were used [16–18]. In the cultured T_CM_ model, T_N_ cells are activated and polarized to direct differentiation into T_CM_ cells, which are phenotypically very similar to T_CM_ cells freshly isolated from blood [17]. Latency is established in the presence of IL-2 and cART in cultured T_CM_ cells that are gradually returning to quiescence and becoming resting; positive magnetic isolation of CD4+ T cells is used to sort out cells that remain productively infected [16, 59]. In the bystander model developed by Dr. Celsa Spina [18, 19], latency is established directly in resting CD4+ T-cells, represented by all maturation phenotypes found *in vivo*. Both the cultured T_CM_ and the bystander models utilized wild type HIV_NL4.3_ virus. Expression of p24/Gag protein during model-set up was assessed as described previously, using ICp24 antibody conjugated to fluorescein isothiocyanate (FITC) (clone KC57, Coulter) for cultured T_CM_ model [16]; for bystander model, ICp24 antibody conjugated to phycoerythrin (PE) (clone KC57, Coulter) was used. To establish a mock-infected control, a portion of cells was cultured under the same conditions in parallel, but without exposure to the virus.

### Identification of lncRNAs dysregulated in HIV latency

For the cultured T_CM_ model, we used a previously published dataset [21] (raw and processed data available at Gene Expression Omnibus (GEO) database, accession number https://www.ncbi.nlm.nih.gov/geo/query/acc.cgi?acc=GSE81810) to re-analyze focusing on dysregulated lncRNAs. For the bystander model, we used RNA-Seq data available at GEO under accession number GSE114883. This dataset includes samples for mock-infected cells and latency model treated with HDACi SAHA and RMD and their solvent dimethyl sulfoxide (DMSO). DMSO-treated samples from this dataset were used to perform differential gene expression analysis between mock-infected cells and cells from the model of HIV latency. To minimize variation due to analytical procedures, bystander model data was analyzed in the same manner as previously published T_CM_ model dataset [21]. Briefly, Tophat [66] was used for mapping to human genome version hg38 and HTSeq [67] for read counting against the Gencode gene annotation version 21. Mapping to HIV and synthetic RNA standards from the External RNA Controls Consortium (ERCC) was performed using Bowtie [68]. Differential expression analysis was performed using library *EdgeR* [69] in Bioconductor R and normalization to ERCC spike-ins with RUVSeq [70]. Genes and lncRNAs with false discovery rate (FDR) corrected *p*-values less than 0.05 were considered differentially expressed.

Among total differentially expressed genes in both models, lncRNAs and their types (intergenic, intronic, etc.) were determined using Gencode gene annotation version 21 with Ensembl IDs as unique identifiers. Visualization of differential expression included volcano plots and heatmaps that were constructed via in-house bioinformatics scripts and based on the package *gplots* (v3.0.1) in the R computing environment [71]. Enrichment of lncRNA types in the lists of dysregulated lncRNAs was evaluated via comparison of ratios with the lncRNA type distribution of the total pool of lncRNAs in the Gencode database (Fisher’s exact test).

### Isolation of T_N_, T_CM_, and T_EM_ CD4+ T-cell subsets

All antibodies were obtained from BD Biosciences, Inc. (San Jose, CA, USA). Antibodies for cell quality control were αCD4 (multiclone SK3 SK4) conjugated to FITC, and αHLA-DR (clone L243) conjugated to PE. Antibodies for isolation of CD4+ T-cell maturation subsets were αCD45RA (clone L48) conjugated to phycoerythrin-cyanine dye (PE-Cy7), and αCD62L (clone DREG-56) conjugated to allophycocyanin (APC). CD4+ T-cells were isolated from whole blood using negative selection (StemCell Technologies, Inc., Vancouver, Canada). All CD4+ T-cell samples had >95% purity and <10% expression of activation marker HLA-DR, as assessed using the Accuri C6 flow cytometer (BD Biosciences, Inc., San Jose, CA, USA). To isolate T-cell maturation subsets, CD4+ T-cells were stained with αCD45RA and αCD62L antibodies, and sorted on a MoFlo XDP Cell Sorter (Beckman Coulter, Brea, CA, USA) to obtain T_N_ (CD45RA+CD62L+), T_CM_ (CD45RA-CD62L+) and T_EM_ (CD45RA-CD62L-) cell populations.

### Treatment with SAHA and RMD

Aliquots of SAHA and RMD solubilized in DMSO were provided by Merck Research Laboratories, Inc. (Kenilworth, NJ, USA), and Gilead Sciences, Inc. (Foster City, CA, USA), respectively. Mock-infected cells and the bystander model of HIV latency were treated with 1 μM SAHA, 15 nM RMD or their solvent DMSO for 24 hours.

### Isolation of RNA

RNA was isolated from CD4+ T-cells using RNeasy micro kit (Qiagen, Inc., Valencia, CA). RNA concentrations were assessed using Qubit 2.0 fluorometer (Thermo Fisher Scientific, Inc., Waltham, MA, USA).

### Droplet digital PCR (ddPCR)

Four lncRNA dysregulated in both models of latency were selected for confirmation by ddPCR. The selection was made based on expression level likely detectable in a single ddPCR reaction (number of reads per base in the RNA-Seq experiment >0.1). Reads per base were calculated by dividing the number of reads mapped to each lncRNA by the average transcript length as determined by *GenomicFeatures* library [72] in Bioconductor R. The assays to measure lncRNA expression were custom designed and purchased from Integrated DNA Technologies, Inc. (Coralville, IA, USA). The assays were as follows: *NEAT1* both short and long isoforms (N1+N2): forward 5’ TTCATGGACCGTGGTTTG 3’, reverse 5’ 56-FAM/CTGCAATGCTAGGACTCAC 3’, probe 5’ TTCCTCATG/ZEN/GCGAGCAGATGGAAC/3IABkFQ 3’; *NEAT1* long isoform (N2): 5’ forward ACGTGTTGCATGGTTTCT 3’, reverse: 5’ ATGAGGGCAGTTCTCTGT 3’, probe 5’ 56-FAM/AACAGTAGG/ZEN/GAGATGCCTGGGAGTA/3IABkFQ 3’; *PVT1*: forward 5’ GAGGGTTGAGATCTCTGTTTAC 3’, reverse 5’ GATGCTTCACCAGGAAGAG 3’, probe 5’ 56-FAM/TCTGCCAAC/ZEN/TTCCTTTGGGTCTCC/3IABkFQ 3’; *RP11-347C18*.*3*: forward 5’ AGCTCTCATGTGACCCA 3’, reverse 5’ AATAACCTGGTGAGTTGGC 3’, probe 5’ 56-FAM/ACCTGCAAA/ZEN/TTGTGGGCATTCACG/3IABkFQ 3’; *RP11-539L10*.*2*: forward 5’ TTTGGTCCCTGGCTTTG 3’, reverse 5’ CCCTATCTCTGATCATTGTCAC 3’, probe 5’ 56-FAM/TTGTGACCC/ZEN/GAGTGTCAGTTTCCT/3IABkFQ/ 3’. *NRON* lncRNA, which was not annotated in the RNA-Seq datasets, was measured by ddPCR using assay Hs04274937_s1 from Applied Biosystems (now Thermo Fisher Scientific, Inc., Waltham, MA, USA). Housekeeping gene Ribosomal Protein L27 (*RPL27*) was used as a normalizer [55, 58, 73]. The assay (HS03044961_g1) to measure *RPL27* was purchased from Applied Biosystems (now Thermo Fisher Scientific, Inc., Waltham, MA, USA). RNA was converted to cDNA using qScript (Quanta Bio, Beverly, MA). Concentration of RNA in all cDNA reactions was 1 ng/μl, except for lowly expressed *NRON*, for which 10 ng/μl was used. DdPCR reactions were set up and run as described previously [58]. LncRNAs whose dysregulation in latency were confirmed (*PVT1*, *RP11-347C18*.*3*, *RP11-539L10*.*2* and *NRON*) were further measured in CD4+ T-cell maturation subsets and in CD4+ T-cells following treatment with SAHA and RMD.

### GBA analysis

The GBA analysis to associate lncRNAs with biological pathways was performed as described previously [14, 74]. Briefly, the GBA pipeline first builds a Spearman’s rank correlation matrix between the differentially expressed lncRNAs and all expressed mRNAs based on the normalized gene expression values of the samples (counts per million). Next, a ranked mRNA gene list was constructed for each lncRNA based on their correlation coefficients and used as input for gene set enrichment analysis (GSEA) [75] together with pathways from the Biocarta [76] and KEGG [77] databases. A lncRNA was considered significantly associated to a pathway when the GSEA FDR corrected *p*-value was less than 0.05. The GBA was implemented separately for the primary models of HIV latency and results were compared and overlapped afterwards. Visualization of significant pathway- and mRNA-associations was performed with in-house R scripts based on the *ggplot2* package (v2.2.1). Visualization of individual lncRNA associations was performed using Cytoscape v3.4.0 [78].

### Statistical analyses

All statistical analyses were performed in the R computing environment [71]. Effect size in the RNA-Seq experiment was determined using library *RNASeqPower* [79]. DdPCR data was expressed as copies of each lncRNA per one thousand copies of *RPL27*. Because of lower expression of *NRON*, this lncRNA was expressed as copies per hundred thousand copies of *RPL27*. These normalized copies were log_2_ transformed. The equal variance test was performed using function *var*.*test*. Based on these results, paired *t*-tests (function *t*.*test*) with equal or unequal variance were used for two-group comparisons. For ddPCR validation experiments, we had prior knowledge about the direction of change of expression in models of latency as compared to mock-infected cells; therefore, one-sided tests were performed. For two-group comparisons without prior knowledge of the direction of change, two-sided tests were conducted. Repeated measures analysis of variance (RM ANOVA) was implemented for multi-group comparisons using library *nlme* [80] followed by post-hoc Tukey test. For each model, residuals were measured and tested for normality using the shapiro.test in R. Examination of the distribution of residuals and ggplots indicated that there were no large deviations from normality; therefore the requirements of the *t*-test and ANOVA were satisfied. All graphs were constructed using GraphPad Prism software (GraphPad Software, La Jolla, CA, USA). If RNA from the same donor was used for multiple measurements, the symbol for that donor is consistently shaped and colored throughout.

## Supporting information

S1 Fig Comparison of dysregulated protein coding genes and lncRNAs in the cultured T_CM_ and the bystander latency models. Venn diagram was constructed using library VennDiagram v1.16.18 in the R computing environment. Dysregulated lncRNA and protein coding genes from S1 Table served as input for construction of the Venn diagram. Area of the diagram is proportional to the number of differentially expressed lncRNAs or protein coding genes.
(PDF)

S2 Fig Validation of lncRNA dysregulation in latency by ddPCR. Same samples that were sequenced (cultured T_CM_ model, N = 4) and three additional bystander model samples (bystander model, N = 7) were subjected to ddPCR with assays to detect selected lncRNA. Expression of *PVT1*, *RP11-347C18*.*3*, *RP11-539L10*.*2*, *NEAT1* and *NRON* was measured by ddPCR and normalized to expression of the housekeeping gene *RPL27*. Significance for ddPCR results was determined using a paired one-sided *t*-test for log_2_ transformed data. Data is presented as individual data points (copy numbers normalized to *RPL27*) symbol-coded by donor, mean of all values is shown. N1, *NEAT1* isoform 1 (short); N2, *NEAT1* isoform 2 (long); *ns*, not significant (*p*-value > 0.05); (*) represents experiments where for the cultured T_CM_ model only three out of four sequenced sample pairs had sufficient RNA for testing.
(PDF)

S3 Fig Comparison of lncRNA expression in T_N_, T_CM_, and T_EM_ T-cells. Expression of *PVT1*, *RP11-347C18*.*3*, *RP11-539L10*.*2* and *NRON* was measured by ddPCR and normalized to expression of the housekeeping gene *RPL27*. Out of six experiments shown in Fig 2, a subset of replicates had sufficient number of T_EM_ cells to conduct the assays (N = 4 for *PVT1*, *RP11-347C18*.*3* and *RP11-539L10*.*2*; N = 3 for *NRON*). Significance was determined by implementing repeated measures analysis of variance (RM ANOVA) with library *nlme* in R using log_2_ transformed data. Data is presented as individual data points (copy numbers normalized to *RPL27*) symbol-coded by donor, mean of all values is shown. T_N_, naïve CF4+ T-cells; T_CM_, central memory CD4+ T-cells, T_EM_, effector memory CD4+ T-cells.
(PDF)

S4 Fig Unique and common KEGG pathways associated with lncRNA dysregulated in latency for the cultured T_CM_ and the bystander models, identified by the guilt-by-association (GBA) analysis. A. Lists of common and unique pathways. B. Venn diagram showing overlap of pathways between the two models.
(PDF)

S5 Fig Unique and common Biocarta pathways associated with lncRNA dysregulated in latency for the cultured TCM and the bystander models, identified by the guilt-by-association (GBA) analysis. A. Lists of common and unique pathways. B. Venn diagram showing overlap of pathways between the two models.
(PDF)

S6 Fig Down-regulation of *RP11-539L10*.*2* by SAHA and RMD. Mock-infected cells and the bystander model of HIV latency were treated with SAHA (1μM) and RMD (15 nM) or their solvent DMSO for 24 hours. Expression of *RP11-539L10*.*2* was measured by ddPCR and normalized to expression of the housekeeping gene *RPL27*. Four replicate experiments were performed. Significance was determined by implementing repeated measures analysis of variance (RM ANOVA) with library *nlme* in R using log_2_ transformed data. Data is presented as individual data points (copy numbers normalized to *RPL27*) symbol-coded by donor, mean of all values is shown. DMSO, dimethyl sulfoxide; SAHA, suberoylanilide hydroxamic acid; RMD, Romidepsin; *ns*, not significant (*p*-value > 0.05).
(PDF)

S1 Table Genes and lncRNAs dysregulated in latency. Data is organized in two Excel sheets, showing genes and lncRNAs dysregulated in the cultured T_CM_ and the bystander models of HIV latency. *Ensembl_ID*, Ensembl gene identifier; *HGNC_Gene_Symbol*, gene symbol approved by the HUGO gene nomenclature committee (gencode 21); *logFC*, log_2_ transformed fold change of counts per million in the RNA-Seq data; *FDR*, false discovery rate adjusted *p*-value; *gene_type*, class of gene according to the nomenclature (protein coding, etc.). Data is sorted by gene_type and then by FDR within each gene type.
(XLSX)

S2 Table Cellular pathways associated with lncRNAs *RP11-347C18*.*3* and *RP11-539L10*.*2*. Listed are KEGG and Biocarta pathways that were associated (FDR *p*<0.05) with *RP11-347C18*.*3* and *RP11-539L10*.*2* in common for the cultured T_CM_ and the bystander models of HIV latency, and unique pathways found for each model. Pathways implicated in HIV latency are highlighted *red*.
(XLSX)
