# Supplementary figures and images for: Long non-coding RNAs and latent HIV – A search for novel targets for latency reversal
Source: PLoS One. 2019 Nov 11;14(11):e0224879. doi: 10.1371/journal.pone.0224879 (PMC6844474; doi:10.1371/journal.pone.0224879)

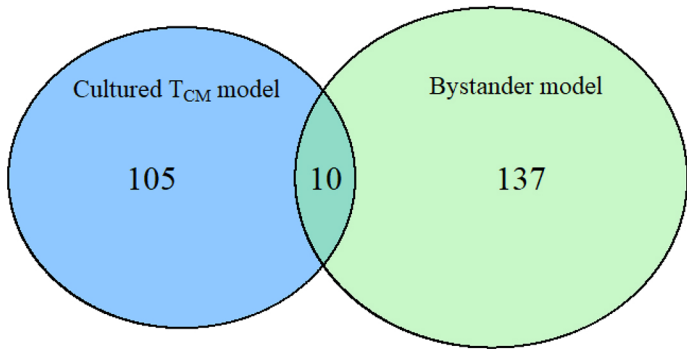

LncRNA

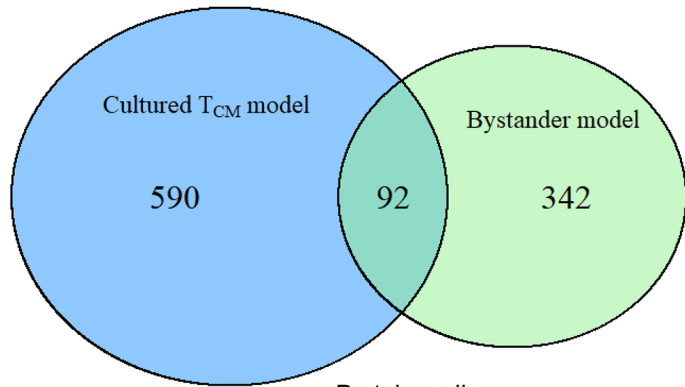

Protein coding

Supplement: S1 Fig — Venn diagram was constructed using library VennDiagram v1.16.18 in the R computing environment. Dysregulated lncRNA and protein coding genes from S1 Table served as input for construction of the Venn diagram. Area of the diagram is proportional to the number of differentially expressed lncRNAs or protein coding genes. (PDF) [file pone.0224879.s001.pdf]

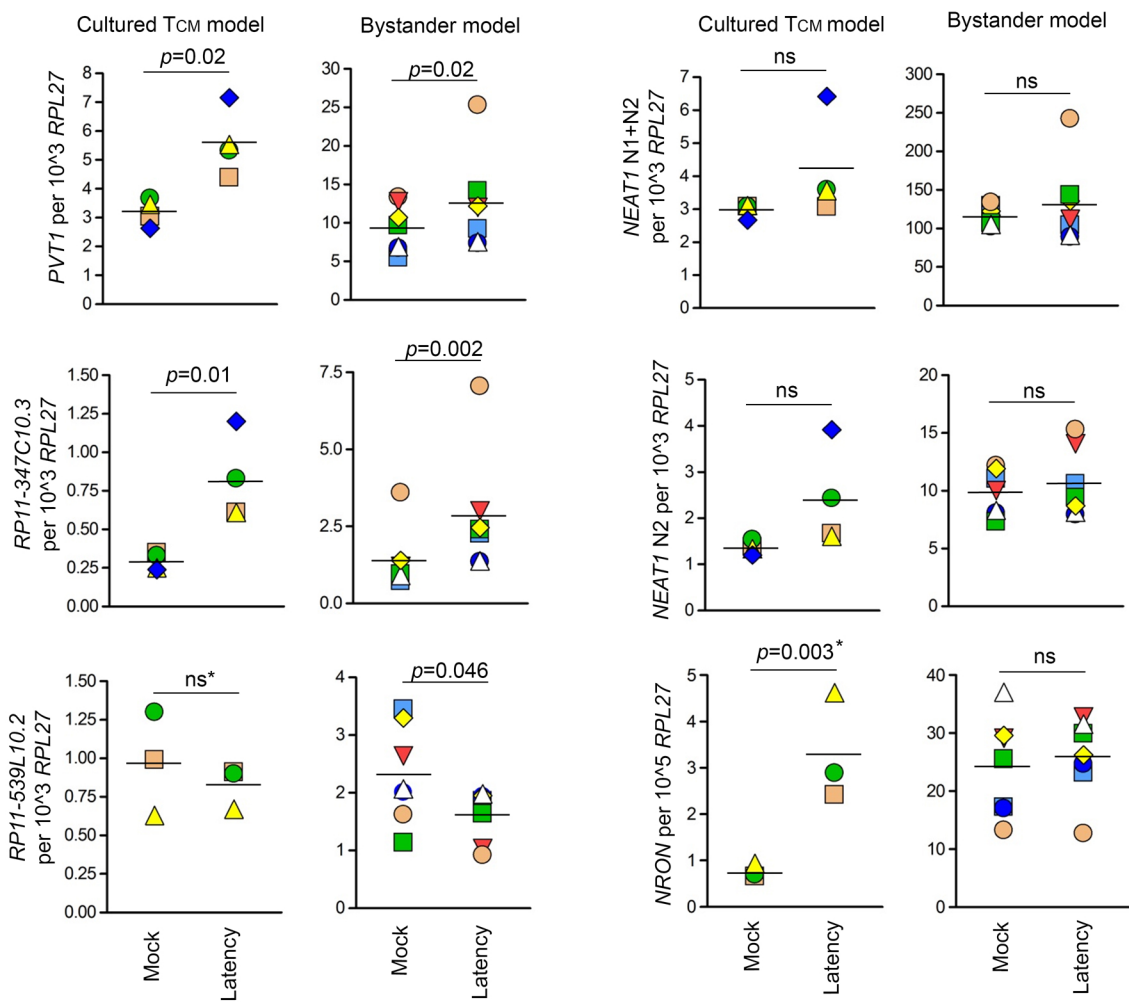

Supplement: S2 Fig — Same samples that were sequenced (cultured TCM model, N = 4) and three additional bystander model samples (bystander model, N = 7) were subjected to ddPCR with assays to detect selected lncRNA. Expression of PVT1, RP11-347C18.3, RP11-539L10.2, NEAT1 and NRON was measured by ddPCR and normalized to expression of the housekeeping gene RPL27. Significance for ddPCR results was determined using a paired one-sided t-test for log2 transformed data. Data is presented as individual data points (copy numbers normalized to RPL27) symbol-coded by donor, mean of all values is shown. N1, NEAT1 isoform 1 (short); N2, NEAT1 isoform 2 (long); ns, not significant (p-value > 0.05); (*) represents experiments where for the cultured TCM model only three out of four sequenced sample pairs had sufficient RNA for testing. (PDF) [file pone.0224879.s002.pdf]

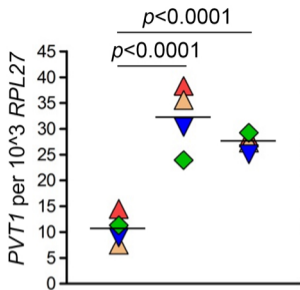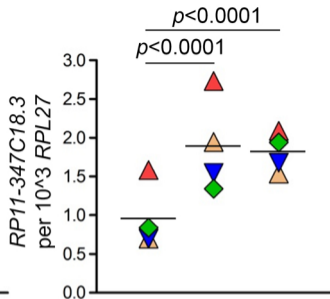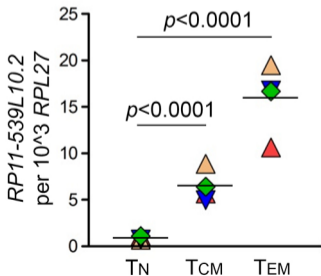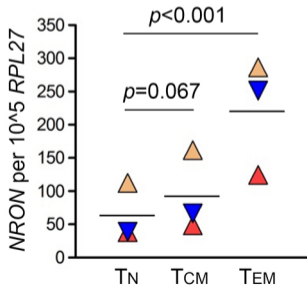

Supplement: S3 Fig — Expression of PVT1, RP11-347C18.3, RP11-539L10.2 and NRON was measured by ddPCR and normalized to expression of the housekeeping gene RPL27. Out of six experiments shown in Fig 2, a subset of replicates had sufficient number of TEM cells to conduct the assays (N = 4 for PVT1, RP11-347C18.3 and RP11-539L10.2; N = 3 for NRON). Significance was determined by implementing repeated measures analysis of variance (RM ANOVA) with library nlme in R using log2 transformed data. Data is presented as individual data points (copy numbers normalized to RPL27) symbol-coded by donor, mean of all values is shown. TN, naïve CF4+ T-cells; TCM, central memory CD4+ T-cells, TEM, effector memory CD4+ T-cells. (PDF) [file pone.0224879.s003.pdf]

# Mock-infected cells

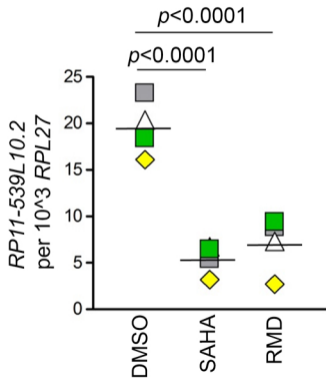

# Bystander model

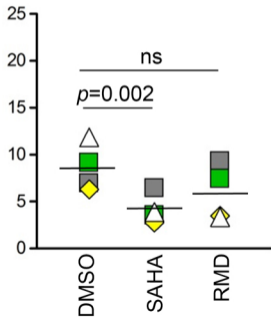

Supplement: S6 Fig — Mock-infected cells and the bystander model of HIV latency were treated with SAHA (1μM) and RMD (15 nM) or their solvent DMSO for 24 hours. Expression of RP11-539L10.2 was measured by ddPCR and normalized to expression of the housekeeping gene RPL27. Four replicate experiments were performed. Significance was determined by implementing repeated measures analysis of variance (RM ANOVA) with library nlme in R using log2 transformed data. Data is presented as individual data points (copy numbers normalized to RPL27) symbol-coded by donor, mean of all values is shown. DMSO, dimethyl sulfoxide; SAHA, suberoylanilide hydroxamic acid; RMD, Romidepsin; ns, not significant (p-value > 0.05). (PDF) [file pone.0224879.s006.pdf]
