# Supplementary material for: Long non-coding RNAs and latent HIV – A search for novel targets for latency reversal
Source: PLoS One. 2019 Nov 11;14(11):e0224879. doi: 10.1371/journal.pone.0224879 (PMC6844474; doi:10.1371/journal.pone.0224879)

A.

Pathways unique for cultured T_{CM} model
ASTHMA
LYSOSOME
PYRIMIDINE_METABOLISM
CELL_CYCLE
NATURAL_KILLER_CELL_MEDIATED_CYTOTOXICITY
SPHINGOLIPID_METABOLISM
VEGF_SIGNALING
PRIMARY_IMMUNODEFICIENCY
ENDOCYTOSIS
N_GLYCAN_BIOSYNTHESIS
PURINE_METABOLISM
DRUG_METABOLISM_OTHER_ENZYMES
FRUCTOSE_AND_MANNOSE_METABOLISM
DORSO_VENTRAL_AXIS_FORMATION
B_CELL_RECEPTOR_SIGNALING
THYROID_CANCER
ALANINE_ASPARTATE_AND_GLUTAMATE_METABOLISM
INOSITOL_PHOSPHATE_METABOLISM
PHOSPHATIDYLINOSITOL_SIGNALING_SYSTEM
LEISHMANIA_INFECTION
ANTIGEN_PROCESSING_AND_PRESENTATION
CYSTEIN_AND_METHIONINE_METABOLISM
ENDOMETRIAL_CANCER
VALINE_LEUCINE_AND_ISOLEUCINE_DEGRADATION
DNA_REPLICATION
AMINO_SUGAR_AND_NUCLEOTIDE_SUGAR_METABOLISM
BUTANOATE_METABOLISM
HOMOLOGOUS_RECOMBINATION
GLYCOLYSIS_GLUCONEOGENESIS
PPAR_SIGNALING
REGULATION_OF_ACTIN_CYTOSKELETON
PORPHYRIN_AND_CHLOROPHYLL_METABOLISM
CYTOSOLIC_DNA_SENSING
TRYPTOPHAN_METABOLISM
JAK_STAT_SIGNALING
COLORECTAL_CANCER
BASAL_TRANSCRIPTION_FACTORS
NUCLEOTIDE_EXCISION_REPAIR
PROGESTERONE_MEDIATED_OOCYTE_MATURATION
CHEMOKINE_SIGNALING
HEDGEHOG_SIGNALING

Common pathways for cultured T_{CM} and bystander models
RIBOSOME
SPLICEOSOME
SYSTEMIC_LUPUS_ERYTHEMATOSUS
GLYCOSAMINEGLYCAN_BIOSYNTHESIS_HEPARAN_ SULPHATE
CYTOKINE_CYTOKINE_RECEPTOR_INTERACTION
CELL_ADHESION_MOLECULES_CAMS
FATTY_ACID_METABOLISM
INTESTINAL_IMMUNE_NETWORK_FOR_IGA_PRODUCTION
TYPE_I_DIABETES_MELLITUS
ALLOGRAFT_REJECTION
COMPLEMENT_AND_COAGULATION_CASCADES
GRAFT_VERSUS_HOST_DISEASE
AUTOIMMUNE_THYROID_DISEASE
PATHOGENIC_ESCHERICHIA_COLI_INFECTION
AMINOACYL_TRNA_BIOSYNTHESIS
RNA_DEGRADATION
RNA_POLYMERASE
GLYCOSAMINEGLYCAN_BIOSYNTHESIS_CHONDROITIN_ SULPHATE
P53_SIGNALING
STARCH_AND_SUCROSE_METABOLISM
PROTEIN_EXPORT
PROTEASOME
HEMATOPOIETIC_CELL_LINEAGE
GLUTATHIONE_METABOLISM
HYPERTROPHIC_CARDIOMYOPATHY_HCM
VIRAL_MYOCARDITIS
OCYTE_MEIOISIS
STEROID_BIOSYNTHESIS
PARKINSONS_DISEASE
PEROXISOME
GALACTOSE_METABOLISM
CARDIAC_MUSCLE_CONTRACTION
OXIDATIVE_PHOSPHORYLATION
CITRATE_CYCLE_TCA_CYCLE
BASAL_CELL_CARCINOMA
METABOLISM_OF_XENOBIOTICS_BY_CYTOCHROME_P450
TGF_BETA_SIGNALING
ALZHEIMERS_DISEASE
LEUKOCYTE_TRANSENDOTHELIAL_MIGRATION

Pathways unique for bystander model
GLYCEROPHOSPHOLIPID_METABOLISM
UBIQUITIM_MEDIATED_PROTEOLYSIS
PENTOSE_PHOSPHATE
NOTCH_SIGNALING
ABC_TRANSPORTERS
DILATED_CARDIOMYOPATHY
ECM_RECEPTOR_INTERACTION
HUNTINGTONS_DISEASE
MTOR_SIGNALING
WNT_SIGNALING
GLYCOSAMINOGLYCAN_DEGRADATION
ONE_CARBON_POOL_BY_FOLATE
ARACHIDONIC_ACID_METABOLISM
PYRUVATE_METABOLISM
MISMATCH_REPAIR
ARGININE_AND_PROLINE_METABOLISM
BASE_EXCISION_REPAIR
GLYCOSPHINGOLIPID_BIOSYNTHESIS_LACTO_AND_ NEOLACTO_SERIES
GLYCINE_SERINE_AND_THREONINE_METABOLISM
O_GLYCAN_BIOSYNTHESIS

B.

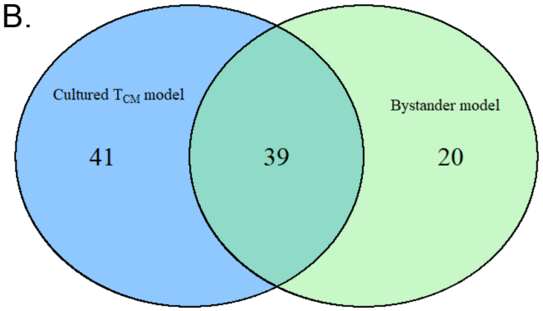

Supplement: S4 Fig — A. Lists of common and unique pathways. B. Venn diagram showing overlap of pathways between the two models. (PDF) [file pone.0224879.s004.pdf]
