# Supplementary material for: Long non-coding RNAs and latent HIV – A search for novel targets for latency reversal
Source: PLoS One. 2019 Nov 11;14(11):e0224879. doi: 10.1371/journal.pone.0224879 (PMC6844474; doi:10.1371/journal.pone.0224879)

A.

Pathways unique for cultured T_{CM} model
VEGF
TH1TH2
INTEGRIN
G2
ATRBRCA
IL10
BIOPEPTIDES
MCM
RACCYCD
G1
CELLCYCLE
TNFR1
STRESS
NFKB
STATHMIN
CHREBP2
HER2
IL2RB
CD40
TID
CDMAC
TPO
EGF
PDGF
CCR5
GPCR
AT1R
IL2
EIF4
WNT
TEL

Common pathways for cultured T_{CM} and Spina models
MCALPAIN
TOB1
TGFB
ATM
RHO
CDC42RAC
MPR
PROTEASOME
BCELLSURVIVAL
EDG1
IL7
ARAP
TNFR2
GCR
NGF
TCR
EPO
IL6
INSULIN
EIF
MTOR
IGF1MTOR

Pathways unique for bystander model
RAC1
MITOCHONDRIA
DEATH
NKT
VIP
RELA
NTHI
ETS
RARRXR
CARM_ER
FMLP
SPPA
ERK
PML
P53HYPOXIA
P53
BAD
IGF1R
CREB
GLEEVEC
TFF
ERK5
PTEN
RAS
AKT
CTLA4
CSK
ACTINY

B.

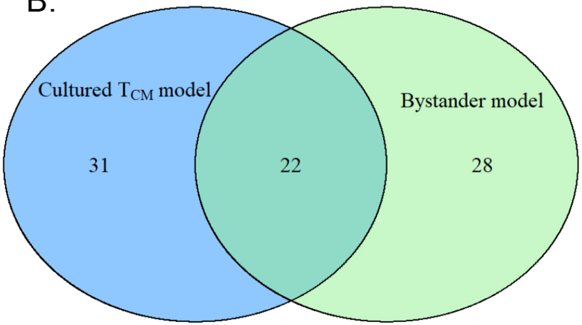

Supplement: S5 Fig — A. Lists of common and unique pathways. B. Venn diagram showing overlap of pathways between the two models. (PDF) [file pone.0224879.s005.pdf]
